# Polarity-Dependent Transcranial Direct Current Stimulation Effects on Central Auditory Processing

**DOI:** 10.1371/journal.pone.0025399

**Published:** 2011-09-23

**Authors:** Andrea Ladeira, Felipe Fregni, Camila Campanhã, Cláudia Aparecida Valasek, Dirk De Ridder, André Russwsky Brunoni, Paulo Sérgio Boggio

**Affiliations:** 1 Social and Cognitive Neuroscience Laboratory and Developmental Disorders Program, Center for Health and Biological Sciences, Mackenzie Presbyterian University, Sao Paulo, Brazil; 2 Laboratory of Neuromodulation, Spaulding Rehabilitation Hospital and Massachusetts General Hospital, Harvard Medical School, Boston, Massachusetts, United States of America; 3 Berenson-Allen Center for Noninvasive Brain Stimulation, Beth Israel Deaconess Medical Center, Harvard Medical School, Boston, Massachusetts, United States of America; 4 BRAI2N/TRI and Department of Neurosurgery, University Hospital Antwerp, Antwerp, Belgium; 5 Núcleo de Neurociências e Comportamento, Instituto de Psicologia, Universidade de São Paulo, São Paulo, Brazil; University of Salamanca-Medical School, Spain

## Abstract

Given the polarity dependent effects of transcranial direct current stimulation (tDCS) in facilitating or inhibiting neuronal processing, and tDCS effects on pitch perception, we tested the effects of tDCS on temporal aspects of auditory processing. We aimed to change baseline activity of the auditory cortex using tDCS as to modulate temporal aspects of auditory processing in healthy subjects without hearing impairment. Eleven subjects received 2mA bilateral anodal, cathodal and sham tDCS over auditory cortex in a randomized and counterbalanced order. Subjects were evaluated by the Random Gap Detection Test (RGDT), a test measuring temporal processing abilities in the auditory domain, before and during the stimulation. Statistical analysis revealed a significant interaction effect of time vs. tDCS condition for 4000 Hz and for clicks. Post-hoc tests showed significant differences according to stimulation polarity on RGDT performance: anodal improved 22.5% and cathodal decreased 54.5% subjects' performance, as compared to baseline. For clicks, anodal also increased performance in 29.4% when compared to baseline. tDCS presented polarity-dependent effects on the activity of the auditory cortex, which results in a positive or negative impact in a temporal resolution task performance. These results encourage further studies exploring tDCS in central auditory processing disorders.

## Introduction

The processing of auditory information, an essential component of language, involves a complex neural network [1], [2] composed of auditory pathway structures such as the cochlear nuclei, lateral lemniscus, inferior colliculus, medial geniculate nucleus and superior temporal gyrus. The peripheral system is essential for the accurate auditory sensation or signal detection, whereas structures such as the superior medial and the lateral olivary nuclei are involved with specific aspects of sound localization (intensity and latency, respectively). On the other hand, cortical components such as the superior temporal gyrus are involved in auditory discrimination, temporal aspects of hearing (such as resolution, masking, integration and temporal ordering), recognition of auditory patterns, and auditory performance in the presence of competitive acoustic stimuli [3]. Thus, failure or interference in the cortical processing of auditory information will affect the integration, understanding and, finally, interpretation of sound stimuli.

In this sense, several studies have investigated the negative impact of changes in the central auditory processing in patients with neurological disorders [4], [5], children with learning disabilities [6], [7], [8], and normal aging [9] that result in deficits on speech perception. In this scenario, non-invasive alternatives to modulate specific central auditory functions that ultimately may promote gains in sound processing and speech perception are desirable.

One manner to modulate cortical activity safely and powerfully is using transcranial direct current stimulation (tDCS) – an effective technique of brain modulation that uses weak direct current to change neuronal spontaneous firing [10]. tDCS effects are polarity dependent, i.e., anodal stimulation is related to a cellular membrane depolarization and cathodal with hyperpolarization [11]. Those effects result, respectively, in facilitation or inhibition of neuronal processing and ultimately can modify behavior according to the stimulated area [12]. For instance, several studies have shown significant changes on motor and visual behavior after application of tDCS [13]–[15]. With regard to auditory processing, tDCS applied to the superior temporal gyrus (STG) modulates pitch discrimination [16], [17], in a polarity dependent way: whereas only cathodal transcranial direct current stimulation over the left supramarginal gyrus had a detrimental effect on short-term pitch-memory performance in one study [16]. Cathodal stimulation of the STG on the left and on the right hemispheres adversely affected pitch discrimination in comparison to sham stimulation, with the effect on the right being significantly stronger than on the left. Anodal stimulation on either side had no effect on performance in comparison to sham [17].

Based on the abovementioned modulation of pitch discrimination by tDCS targeting the auditory cortex, we aim to evaluate temporal processing in the auditory domain in healthy subjects without hearing impairment. We choose temporal resolution as the main outcome since it is an important component to a normal linguistic performance and it is involved with cortical auditory activation [18] and we use a technique of cortical modulation as the intervention tool.

In summary, this was a double-blinded, randomized, sham-controlled trial that enrolled 11 healthy, young adults. All subjects received successive blocks of anodal, cathodal or sham stimulation, in a randomized, incomplete counterbalanced order as the number of subjects was not multiple of 3. Two active electrodes were placed over T3 and T4 (EEG 10/20 System, area corresponding to the auditory cortex) and two references were placed over the right deltoid muscle (in anodal stimulation, the anodes were on T3/T4 and the cathodes on the arm; and vice-versa for cathodal stimulation). The primary assessment was the random gap detection test (RGDT), which evaluates temporal auditory resolution and can index primary cortical processing. In our study, RGDT was evaluated at different frequency ranges, from lower (500 Hz) to higher (4000 Hz) frequencies and also clicks (white noise). RGDT is a test in which tones are presented in pairs and the interval between them increases or decreases from 0 to 40 msec. Subjects have to identify when tones (from each pair) are separated in time. The primary outcome parameter is detection threshold, defined as the smallest interval in which the individual identifies two separate tones. Based on the polarity dependent effect of tDCS on neuronal spontaneous firing in which anodal stimulation leads to cellular membrane depolarization and cathodal to hyperpolarization [11], our hypothesis was that anodal stimulation would increase performance on RGDT and, conversely, cathodal stimulation would decrease RGDT performance. We therefore tested tDCS polarity and frequency dependent effects.

## Results

All subjects completed the entire experiment. All subjects tolerated the stimulation well and no side effects were reported. Also, bilateral stimulation was not associated with additional discomfort by subjects.

Initially, we performed a repeated measures ANOVA in which the dependent variable was the RGDT threshold for each frequency and the independent variables were: main effects of condition of stimulation (anodal, cathodal or sham), gender (male or female), time (pre and during tDCS), and the following interaction terms: gender×time, gender×tDCS, time×tDCS, and tDCS×gender×time. ANOVA did not reveal any significant effect for 500 Hz, 1000 Hz, and 2000 Hz.

**Figure 1:**
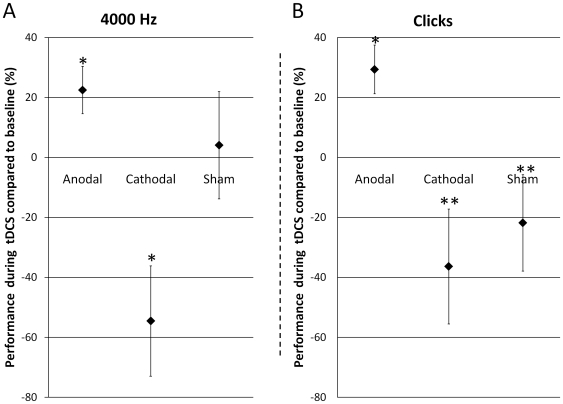
RGDT performance considering time and the type of stimulation (mean ±SEM). 1A presents performance during 4000 Hz. * p = 0.04 for the comparison between performance during anodal tDCS and anodal baseline and between performance during cathodal tDCS and cathodal baseline. 1B presents performance during Clicks. * p = 0.015 for the comparison between performance during anodal tDCS and anodal baseline. ** p = 0.002 and p = 0.04 for performance during anodal tDCS in comparison to cathodal and sham tDCS, respectively.

**Figure 2:**
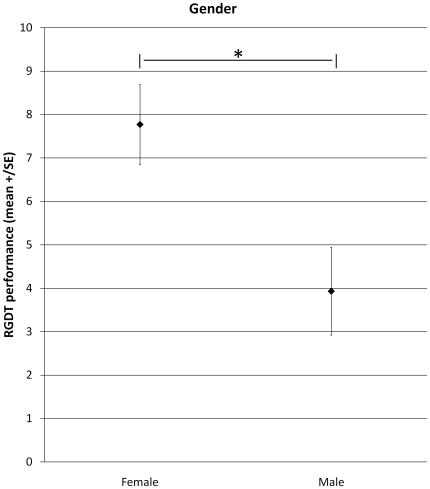
RGDT performance considering gender (mean. ±SEM). * p = 0.02; women presented a worse performance on RGDT in comparison to men for 4000 Hz.

With regard to 4000 Hz, repeated measures ANOVA did not reveal significant effects for tDCS (F_2,18_ = 0.002; p = 1.0), Time (F_1,9_ = 0.6; p = 0.5), and the interaction terms tDCS*Gender (F_2,18_ = 1.2; p = 0.3), Time*Gender (F_1,9_ = 0.00009; p = 1.0), tDCS*Time*Gender (F_2,18_ = 0.6; p = 0.6). However, ANOVA found significant effects for Gender (F_1,9_ = 7.8; p = 0.02) and for the interaction tDCS*Time (F_2,18_ = 5.4; p = 0.01). With regard to the interaction tDCS*Time, Fischer LSD showed significant differences between RGDT performance before anodal tDCS in comparison to RGDT performance during anodal tDCS (p = 0.04); and between RGDT performance before cathodal tDCS in comparison to RGDT performance during cathodal tDCS (p = 0.04). There were no significant effects between the other comparisons. These significant effects, as it can be observed in Figure 1A, were due to an improvement on performance during anodal tDCS (enhancement of 22.5% in comparison to baseline) and a worsening on performance during cathodal tDCS (worsening of 54.5% in comparison to baseline). To exclude a possible effect due to baseline differences between conditions (tDCS groups) we ran a repeated ANOVA on baseline performance considering tDCS as a within-factor. This analysis did not reveal a significant effect between groups at baseline (F_2,20_ = 1.1; p = 0.4). With regard to the main effect of Gender, it was due to a better performance of males as compared to females as it can be seen in Figure 2.

With regard to Clicks, repeated measures ANOVA did not reveal significant effects for Gender (F_1,9_ = 4.9; p = 0.05), tDCS (F_2,18_ = 2.8; p = 0.09), Time (F_1,9_ = 0.0007; p = 1.0), and the interaction terms tDCS*Gender (F_2,18_ = 0.9; p = 0.4), Time*Gender (F_1,9_ = 4.4; p = 0.07), tDCS*Time*Gender (F_2,18_ = 0.5; p = 0.6). However, ANOVA found a significant effect for the interaction tDCS*Time (F_2,18_ = 5.8; p = 0.01). We conducted similar post-hoc comparisons for clicks. This analysis disclosed significant differences in RGDT performance between before vs. during anodal tDCS (p = 0.015). In addition, we observed significant differences in performance between during anodal tDCS vs. cathodal tDCS (p = 0.002) and between during anodal tDCS vs. sham tDCS p = 0.04). These significant effects, as it can be observed in Figure 1B, were due to an improvement on performance during anodal tDCS (enhancement of 29.4% in comparison to baseline). To exclude a possible effect due to baseline differences between conditions (tDCS groups) we ran a repeated ANOVA on the baseline performance considering tDCS as a within-factor. This analysis did not reveal a significant effect between groups at baseline (F_2,20_ = 2.4; p = 0.12).

## Discussion

The main finding of this study was the observed effect of tDCS over auditory cortex in a test of central auditory processing. More specifically, there was a significant effect on the frequency of 4000 Hz and clicks as demonstrated by an improvement in performance during anodal stimulation and performance worsening during cathodal stimulation.

The results observed in our study are compatible with previous observations of polarity-dependent effects of tDCS that were shown in the first tDCS studies indexing cortical excitability via motor cortex stimulation [13], [19], as well as subsequent studies [14], [15], [19]–[23] that showed that direct current stimulation of the visual cortex changes visual-evoked potentials and phosphene detection threshold. Therefore, our study extends previous observations of polarity-specific tDCS physiological effects by showing polarity-specific behavioral changes during auditory cortex stimulation.

One interesting finding is that tDCS effects on auditory processing performance depend on the presented sound frequency. For lower frequencies (500 and 1000 Hz), tDCS induced no significant effects, while for 2000 Hz we observed a tendency towards a significant effect. The impact of tDCS was significant for the highest frequency range (4000 Hz) and also to clicks (white noise). These intriguing findings were unexpected. Considering the tonotopic map of the auditory cortex, possible hypotheses for the observed effects may be raised.

Bhatgnagar [24] and Langers et al., [25] reported that the neurons that respond to the lower frequencies are arranged in the anterolateral position whereas for the higher frequencies they are located in the postero medial part of Heschl's gyrus. Tavalage has demonstrated the existence of at least 4 [26], but most likely 6 [27] tonotopic maps in humans, some on the superior temporal gyrus. Our findings have shown that tDCS was more effective in modulating higher frequencies. One potential explanation is that the positioning of the electrodes was in the posterior portion of the temporal cortex. Previous findings showed that non-invasive stimulation has a major impact on cortical structures under the electrodes area [28]–[30]. Thus, the main effect observed in higher frequency bands can be explained by the positioning of electrodes according to the tonotopic maps - thus, our data agree with the findings of Talavage [26], [27] showing the role of the posterior lateral area in the processing of 4000 Hz bands. Finally, the significant effect on clicks found in our study may be explained by a similar mechanism as complex sounds are processed in a lateral position of the Heschl Gyrus [31]. However, this explanation cannot be fully considered in this experiment considering the lack of a control experiment with the electrodes positioned over anterior and medial areas of the auditory cortex. Therefore, new studies might be performed to understand how specific the effects of tDCS are with regard to electrode placement, i.e. how tDCS can modulate specific frequency bands depending where electrodes are placed. Further studies should explore whether stimulation of anterior areas of the temporal cortex change the performance in lower frequency bands such as 500 and 1000 Hz.

Another possible explanation to our results might be related to the fact that frequencies between 2500 and 4000 Hz (bands in which we found the tDCS effects) are in the range of the most sensitive in humans. Classical studies such the ones made by Fletcher and Munson [32], and Robinson and Dadson [33] and recent findings from Suzuki and Takeshima [34] present data about the equal-loudness-level contours for pure tones. In all these studies, the resultant curves between the sound pressure level and frequency reveals a dip around 4000 Hz. Therefore, our findings in similar frequencies might be due to the fact that this auditory frequency range is more intensively represented in the human cortex.

As we anticipated, there was a difference in performance between men and women regardless of the type of stimulation. The performance of men was better than that observed for women for 4000 Hz. These findings are in line with Zaidan et al. [35] and Samelli's [36] reports which revealed that female subjects presented a worse performance than those of males in temporal resolution tasks such as RGDT and GIN. In addition, Ruytjens et al. [37] found gender differences in cerebral blood flow during exposure to white noise and music.

One important limitation of this study is the number of participants. Even considering that our study had a cross-over design and therefore subjects received all types of tDCS, further studies should consider larger sample sizes. In addition, because multiple comparisons were not fully addressed in our manuscript, it is possible that some of the results might have been due to chance. However, given that we used Fisher LSD only if the ANOVA was significant, we have done only 18 comparisons; therefore, it is possible that no more than one comparison (out of the 5 significant comparisons) would be due to chance. Thus our results should be viewed in light of this limitation and thus be confirmed for further trials. Another limitation of our study is that although there were polarity-specific effects, anodal and cathodal effects at 4000 Hz were only statistically significant in the comparison against baseline (and not to sham performance) and thus we could not exclude that a time-dependent drift was partially responsible for the observed effects. However, it should be underscored that time effect was not significant in the statistical models we used in our analysis and, in addition, there are no effects associated with sham stimulation. Finally, the lack of differences between sham and active conditions may be due to the small sample size due to larger variance between conditions. Nonetheless, future studies are needed to confirm the results of our study. Another potential limitation of this study is that our montage had not been previously tested and therefore it is possible that significant current shunting may occur. Based on our and others experiences using extracephalic montages and a recent modeling study [38]–[41], we do not believe that more shunting might have been a problem; though current distribution may be different when using extracephalic electrodes. Although we showed significant behavioral effects with this montage, further studies need to address current distribution using this montage.

Our results show for the first time that tDCS has a polarity-dependent effect on the temporal processing activity of the auditory cortex resulting in a positive or negative impact during temporal resolution task performance. These results encourage further studies exploring the impact of tDCS in patients with central auditory processing disorders as well as studies assessing long-lasting effects of tDCS on auditory processing.

## Materials and Methods

### Study Design

We conducted a double-blinded, randomized, and sham-controlled experiment to investigate the effects of a single-session of tDCS on a temporal central auditory processing task in healthy volunteers. This study conformed to the ethical standards of the Declaration of Helsinki and was approved by the institutional ethics committee from Mackenzie Presbyterian University, Brazil and also by the National Ethics Committee (SISNEP, Brazil - http://portal.saude.gov.br/sisnep).

### Participants

Eleven subjects (5 men; mean age of 21.36±1.03 years) were recruited from Mackenzie Presbyterian University to participate in this study. Written advertisements were posted around campus and interested subjects contacted the study coordinator to enroll. The study coordinator explained the risk/benefits of the study and screened interested individuals for eligibility. Subjects were regarded as suitable to participate in this study if they fulfilled the following criteria: 1) age between 20 and 25 years; 2) no clinically significant or unstable medical, or neuropsychiatric disorder; 3) no history of substance abuse or dependence; 4) no use of central nervous system-affecting medication; 5) no history of brain surgery, tumor, or intracranial metal implantation; 6) Portuguese native speakers; 7) no history of auditory deficits. All subjects were evaluated by a speech therapist and were included in this protocol only if presented normal hearing as assessed by an audiological assessment (0 to 20 dB HL). All subjects were naïve to tDCS and to the Random Gap Detection Test (RGDT). All study participants provided written, informed consent.

If the subject was eligible to participate in this study, he/she would receive anodal, cathodal or sham tDCS (as described below), in a randomized, incomplete counterbalanced order as the number of subjects was not multiple of 3 (the distribution was done using Latin Square Method). TDCS sessions were conducted at the same time on different days with a minimum interval between sessions of 48 hours. The effects of tDCS were measured by RGDT performance - conducted twice for each tDCS session - immediately before and during tDCS.

### Transcranial Direct Current Stimulation (tDCS)

tDCS is based on the application of a weak direct current to the scalp via two saline-soaked surface sponge electrodes and delivered by a battery-driven, constant current stimulator. The device used, developed by our group, is particularly reliable for double-blind studies: a switch can be activated to interrupt the electrical current while maintaining the ON display and showing the stimulation parameters throughout the procedure to the experimenter and participant. Although there is significant shunting of current in the scalp, sufficient current penetrates the brain to modify the transmembrane neuronal potential [28], [29], thus, influencing the level of excitability and modulating the firing rate of individual neurons. The effects on cortical excitability depend on current orientation, such that anodal stimulation generally increases cortical excitability, while cathodal stimulation decreases it [13]. The polarity-specific effects are particularly well-described for motor and visual cortex stimulation.

All subjects received one session per visit of either sham, anodal or cathodal stimulation of the auditory cortex (AC) in a randomized and incomplete counterbalanced order. Two pairs of surface sponge electrodes (35 cm^2^) were soaked in saline and applied to the scalp at the desired sites of stimulation and to the right deltoid muscle as the reference electrode. Rubber bandages were used to hold the electrodes in place for the duration of stimulation. For anodal stimulation of AC, two anode electrodes were placed over T3 and T4 according to the 10–20 system for EEG electrode placement. The reference cathode electrodes were placed over the right deltoid muscle. For cathodal stimulation of AC, two cathode electrodes were placed over T3 and T4 according to the 10–20 system for EEG electrode placement. The reference anode electrodes were placed over the right deltoid muscle. For sham stimulation, the electrodes were placed in the same position, but the stimulator was turned off after 30 seconds of stimulation as previously described being a reliable method of blinding [42].

The rationale for the choice of bilateral anodal or cathodal A1 stimulation was due to our temporal resolution task that assessed both ears simultaneously and also because we were interested to investigate the effects of facilitation or inhibition in auditory temporal processing. In addition, we used an electrode montage with a non-cephalic reference electrode as proposed by other studies [43], [44]. With this montage, we eliminated the confounding effect of the reference electrode.

A constant current of 2 mA was applied for 10 minutes (3 min of tDCS only, and 7 min of tDCS and RGDT).

### Hearing assessment

The audiological evaluation consisted of the following steps: clinical interview, physical examination, tests of middle ear function, pure-tone audiometry, and speech audiometry.

### Central Auditory Processing Task: Random Gap Detection Test (RGDT)

RGDT [45], [46] is a test in which tones are presented in pairs and the interval between them increases or decreases from 0 to 40 msec (in randomized order). Subjects have to identify when tones (from each pair) are separated in time. The threshold of detection is defined as the smallest interval in which the individual identifies two separate tones. The test was developed to measure one aspect of hearing called temporal resolution by determining the smallest interval between two presented stimuli. This range is called the Threshold of Detection of Gap. RGDT is seen as a test to assess the level of integrity in the temporal cortex and was designed to identify disorders of temporal processing that may be related to phonological processing deficits in auditory discrimination of receptive language and reading. Despite being an activity measure of cortical processing, the test has a low cognitive and linguistic load.

RGDT consists on a subtest of practice and four subtests in the frequencies of 500, 1000, 2000 and 4000 Hz of 7 msec long. A final subtest includes a randomized test of clicks (white noisy). Clicks and tones are presented with the following intervals: 0, 2, 5, 10, 15, 20, 25, 30, and 40 msec. The inter-stimulus intervals are recorded with randomized gaps. The pairs of stimuli are presented at intervals of 4–5 seconds so that the individual has time to respond. This test is applied at a comfortable intensity (around 40 dB above the average of hearing thresholds at 500, 1000 and 2000 Hz, and 4000 Hz). Frequency presentation order was randomized across sessions.

The score for this test is based on the definition of the threshold, which is defined as the point where a stimulus is perceived 50% of the time. We recorded the thresholds for each frequency and click before and during each tDCS session. All sessions (tDCS and temporal resolution test) were performed in a sound booth calibrated according to ANSI S3.1-1991. RGDT was administered with a CD-player connected to an audiometer (Maico MA52); throughout the sessions, subjects stayed inside the sound booth with the tDCS electrodes placed on the target areas and using headphones to perform the task.

### Statistical Analysis

Analyses were done with Statistica software (version 8.0, Stat- Soft Inc.). RGDT provides recognition measures of acoustic patterns and the results of this test are indexed by the lower interval of detection time (threshold which is defined as the point where a stimulus is perceived 50% of the time). The task was performed before and during tDCS (anodal, cathodal or sham stimulation) for frequencies of 500 Hz, 1000 Hz, 2000 Hz, 4000 Hz, and clicks (white noise). We performed repeated measures ANOVA in which the dependent variable was the RGDT threshold for each frequency and the independent variables were: main effects of condition of stimulation (anodal, cathodal or sham), gender (male or female), time (pre and during tDCS), and the following interaction terms: gender×time, gender×tDCS, time×tDCS, and tDCS×gender×time. When appropriate, *post-hoc* comparisons were carried out using Fisher's LSD. Unless stated otherwise, all results are presented as means, confidence intervals, and standard errors. Statistical significance refers to a *p* value<0.05.
